# Novel genotypes and phenotypes among Chinese patients with Floating-Harbor syndrome

**DOI:** 10.1186/s13023-019-1111-8

**Published:** 2019-06-14

**Authors:** Shujie Zhang, Shaoke Chen, Haisong Qin, Haiming Yuan, Yalei Pi, Yu Yang, Hui Huang, Guimei Li, Yan Sun, Zhihua Wang, Huamei Ma, Xiaoling Fu, Ting Zhou, Jian Wang, Huifeng Zhang, Yiping Shen

**Affiliations:** 1 Department of Medical Genetics and Molecular Diagnostic Laboratory, Shanghai Children’s Medical Center, Shanghai Jiao Tong University School of Medicine, Shanghai, 200000 People’s Republic of China; 2 Department of Genetics and Metabolism, Maternal and Child Health Hospital of Guangxi Zhuang Autonomous Region, Nanning, 530003 People’s Republic of China; 3 Boston Children’s Hospital, Harvard Medical School, Boston, MA 02115 USA; 4 Dongguan Maternal and Child Health Care Hospital, Dongguan, 523120 People’s Republic of China; 5 Department of pediatrics, The Second Hospital of Hebei Medical University, Shijiazhuang, 050000 People’s Republic of China; 6 Department of Endocrinology, Metabolism, and Genetics, Jiangxi Provincial Children’s Hospital, Nanchang, 330006 People’s Republic of China; 7 Department of Pediatrics Endocrinology, Shandong Provincial Hospital Affiliated to Shandong University, Jinan, 250021 People’s Republic of China; 8 Department of Endocrinology, Genetics and Metabolism, Xi’an Children’s Hospital Affiliated with the School of Medicine, Xi’an Jiaotong University, Xi’an, 710000 People’s Republic of China; 9 Department of Pediatrics, The First Affiliated Hospital, Sun Yat-Sen University, Guangzhou, 510080 People’s Republic of China; 10 Department of Pediatrics, The Peoples Hospital of The Guizhou Province, Guiyang, 550002 People’s Republic of China; 11 Department of Endocrinology, Children’s Hospital of Chongqing Medical University, Chongqing, 400014 People’s Republic of China

**Keywords:** Floating-Harbor syndrome, *SRCAP*, Chinese, Short stature, Growth hormone deficiency

## Abstract

**Background:**

Floating-Harbor syndrome (FHS) is a rare syndromic short stature disorder caused by truncating variants in *SRCAP*. Few Chinese FHS patients had been reported so far and limited knowledge regarding the benefit of growth hormone treatment existed.

**Methods:**

We ascertained 12 short stature patients with molecularly confirmed diagnosis of FHS by whole exome sequencing. We performed a comprehensive clinical evaluation for all patients and assessed the responsiveness of growth hormone treatment in a subset of the patients.

**Results:**

Five distinct pathogenic/likely pathogenic variants were identified in 12 independent FHS patients including two previously reported variants (c.7303C > T/p.Arg2435Ter and c.7330C > T/p.Arg2444Ter) and three novel variants (c.7189G > T/p.Glu2397Ter, c.7245_7246delAT/p.Ser2416ArgfsTer26 and c.7466C > G/p.Ser2489Ter). The c.7303C > T/p.Arg2435Ter mutation appears more common in Chinese FHS patients. The clinical presentations of Chinese FHS patients are very similar to those of previously reported patients of different ethnicities. Yet we noticed micropenis and ear abnormalities in multiple patients, suggesting that these may be novel phenotypes of Floating-Harbor syndrome. Eight patients (one with GH deficiency, one with undetermined GH level, six without GH deficiency) underwent growth hormone treatment, 3 patients had good responses, one with modest and two with poor responses.

**Conclusion:**

We described novel genotypes and phenotypes in a Chinese FHS patient cohort. We showed that about half of FHS patients exhibited modest to good response to GH treatment regardless of their respective GH deficiency status. We didn’t find any correlation between different mutations and response to GH treatment.

**Electronic supplementary material:**

The online version of this article (10.1186/s13023-019-1111-8) contains supplementary material, which is available to authorized users.

## Background

Floating-Harbor syndrome (FHS [MIM 136140]) is a rare condition characterized by short stature, delayed bone age, speech impairment, mild to moderate intellectual disability and distinctive dysmorphic facial features [1, 2]. The typical facial dysmorphic features include triangular face, deep-set eyes, long eyelashes, prominent nose, short philtrum, wide and low-hanging collumella, wide mouth with a thin vermilion border of the upper lip and low-set ears [3, 4]. Just over 100 FHS cases had been reported worldwide [4–8]. Most are sporadic cases occasionally with parent-to-child transmission [4, 9–11]. Truncating mutations in *SRCAP*, which is an SNF2-related chromatin-remodeling factor that serves as a coactivator for CREB-binding protein (CREBBP, better known as CBP, the major cause of Rubinstein-Taybi syndrome [RTS]) had been reported as causal, and the majority of mutations occurred between codon 2407 and 2517 in exon 34 resulting in loss of three C-terminal AT-hook motifs [7, 12]. So far 44 pathogenic variants had been reported in the BIOBASE Human Gene Mutation Database (accessed by Apr. 2018). Among those mutations, c.7330C > T/p.Arg2444Ter is the most frequent mutation in Western FHS individuals and c.7303C > T/p.Arg2435Ter is the second recurrent mutation. Due to limited distribution of pathogenic variants in *SRCAP*, Nikkel S.M. et al. recommended sequencing of *SRCAP* exons 31–34 in all suspected cases to confirm the diagnosis [7]. The overlapping clinical presentations of FHS with other genetic conditions such as Rubinstein-Taybi syndrome, Silver-Russell syndrome, 3 M syndrome and Velo-cardio-facial syndrome had been highlighted by previous studies [13]. The main features of FHS including short stature, delayed bone age and language delay are non-specific and if the facial features are not distinct, the clinical diagnosis can be difficult. A long-term follow-up showed some FHS patients did not fit the classical description and likely had a different condition, which emphasized the importance of molecular diagnosis for appropriate medical intervention [14].

At present, most FHS cases were reported in Western populations including French, Caucasian, Spanish, German, Brazilian, Polish, Finnish and Italian, few FHS patients had been diagnosed in Chinese. In this study, we reported 12 Chinese FHS patients identified by whole exome sequencing (WES) from multiple medical institutions.

## Results

### Clinical phenotypes

**Fig. 1:**
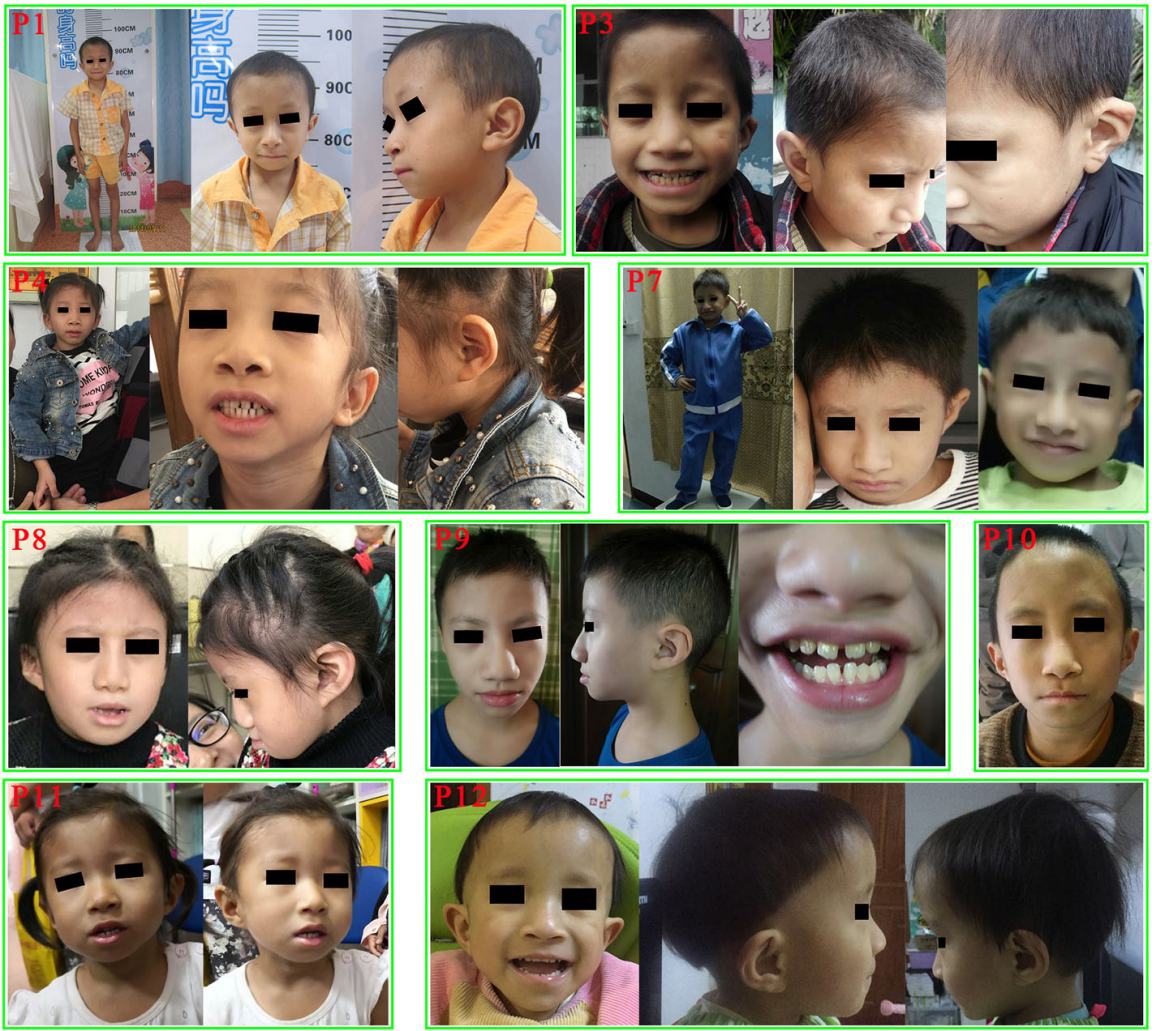
Facial features of nine Chinese patients with FHS. Noticeable features included triangular face, long eyelashes, large and low-set ears, ear deformities, prominent nose, large nares, low-hanging columella, short philtrum, thin vermilion border of the upper lip and small teeth and/or widely spaced teeth

**Fig. 2:**
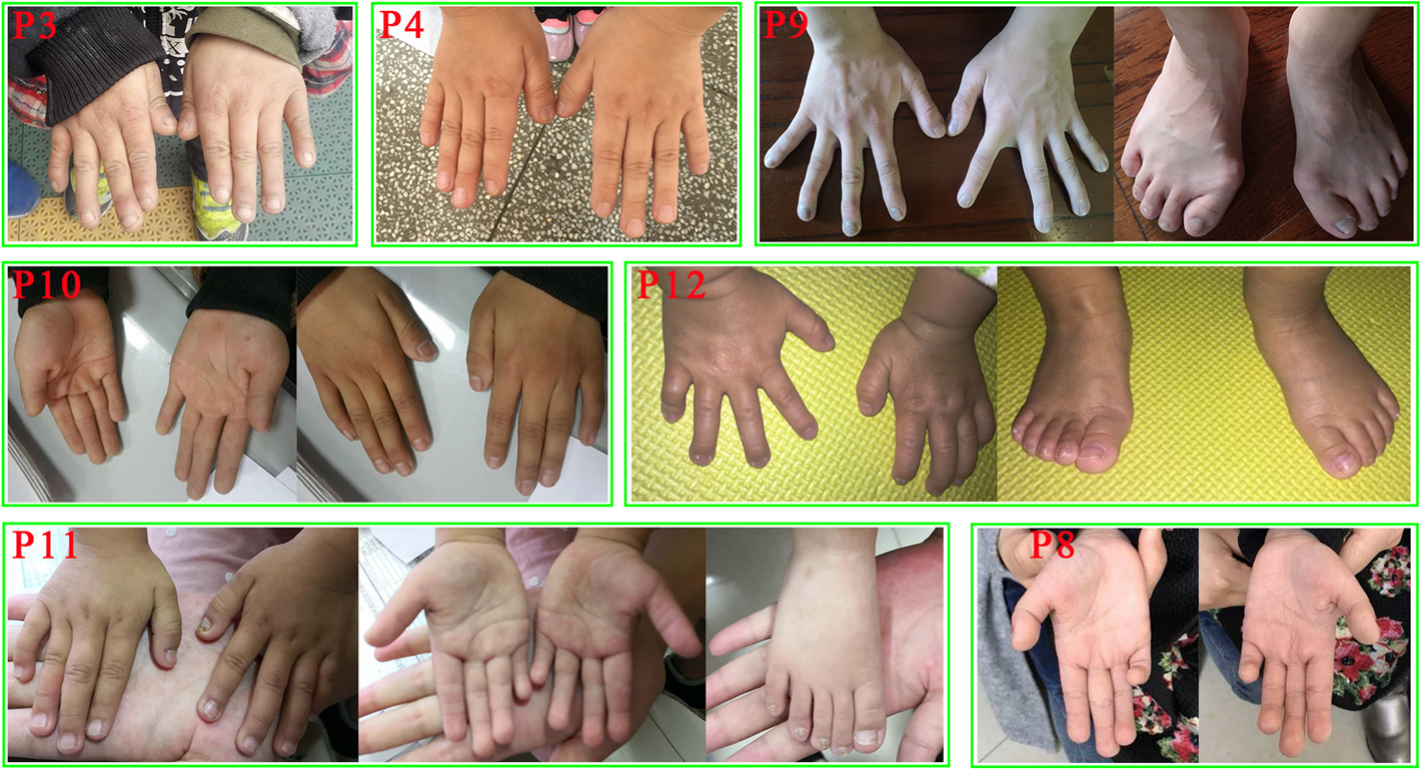
Hands and feet of seven Chinese patients with FHS. These photos showing brachydactyly, broad toes and thumbs, clubbing fingers, broad fingertips, the fourth and fifth fingers clinodactyly and small toenails

**Fig. 3:**
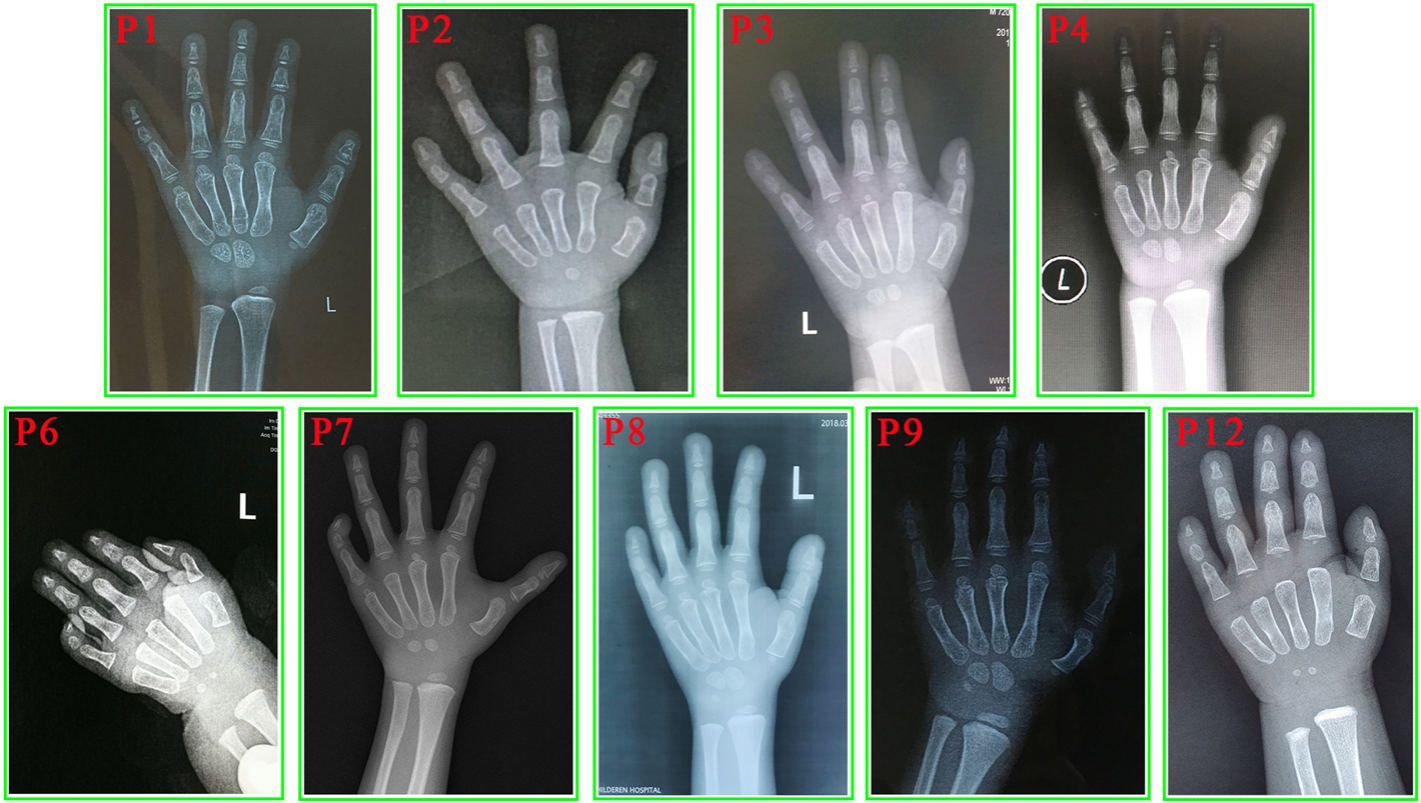
The wrist X-ray results of nine patients with FHS. These photos indicating the bone age delay in each patient. The chronological ages and corresponding bone ages are listed in Table 1

**Fig. 4:**
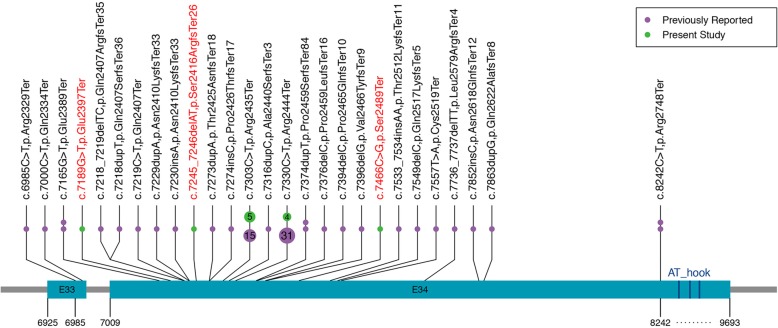
The Summary of reported mutations and novel mutations in FHS patients. Green dots represent mutations found in our cohort; Purple dots represent mutations previously reported in literature. Each point represent one case. The number in circle represent the times of mutations reported. The mutations marked with red color are novel mutations. Three deep blue bars represent three C-terminal AT-hook motifs. The coordinates refer to *SRCAP* cDNA sequence positions

Twelve Chinese FHS patients (6 males, 6 females), all diagnosed by WES, are included in this study. The age of initial assessment was from one year to nine years and two months and the mean age of diagnosis was 3.58 years. Their clinical phenotypes are summarized in Table 1 (details in Additional file 2: Table S1) and presented in Figs. 1, 2 and 3. We reviewed all FHS patients previously reported and only included molecularly confirmed FHS cases for assessing the similarity between Chinese FHS patients and those of other ethnicities (Table 2 and Fig. 4) [5–8, 15–21].

**Table 1:** Summary of clinical and molecular data of 12 FHS patients in our cohort

	Patient 1	Patient 2	Patient 3	Patient 4	Patient 5	Patient 6	Patient 7	Patient 8	Patient 9	Patient 10	Patient 11	Patient 12
*SRCAP* Mutation	c.7303C > T /p.Arg2435Ter	c.7189G > T /p.Glu2397Ter	c.7330C > T /p.Arg2444Ter	c.7303C > T /p.Arg2435Ter	c.7303C > T /p.Arg2435Ter	c.7330C > T /p.Arg2444Ter	c.7245_7246delAT/p.Ser2416ArgfsTer26	c.7330C > T /p.Arg2444Ter	c.7330C > T /p.Arg2444Ter	c.7303C > T /p.Arg2435Ter	c.7303C > T /p.Arg2435Ter	c.7466C > G /p.Ser2489Ter
Exon	E34	E34	E34	E34	E34	E34	E34	E34	E34	E34	E34	E34
*De novo*	Yes	Yes	Yes	Yes	Yes	Yes	Yes	Yes	Yes	NA	Yes	Yes
Short Stature	Yes	Yes	Yes	Yes	Yes	Yes	Yes	Yes	Yes	Yes	Yes	Yes
Age at first assessment	9Y2M	2Y2M	5Y2M	5Y6M	1Y6M	2Y	1Y	5Y	6Y6M	5Y2M	1Y3 M	2Y2M
Delayed Bone Age	Yes	Yes	Yes	Yes	NA	Yes	Yes	Yes	Yes	Yes	NA	Yes
Triangular Face	Yes	Yes		Yes		Yes	Yes	Yes		Yes	Yes	
Low-hanging Columella	Yes	Yes					Yes	Yes		Yes	Yes	Yes
Short Philtrum	Yes						Yes	Yes	Yes	Yes	Yes	Yes
Thin Upper Vermilion Border	Yes	Yes					Yes	Yes	Yes	Yes	Yes	Yes
Wide Mouth		Yes							Yes	Yes		
Deep-set Eyes		Yes			Yes			Yes	Yes			
Long Eyelashes	Yes		Yes		Yes	Yes		Yes	Yes	Yes	Yes	Yes
Low Set Ears	Yes			Yes	Yes			Yes		Yes	Yes	Yes
Broad Thumbs			Yes					Yes	Yes	Yes	Yes	Yes
Brachydactyly			Yes	Yes		Yes		Yes	Yes	Yes	Yes	Yes
Clinodactyly V finger					Yes	Yes		Yes	Yes			
Small Teeth/Widely Spaced Teeth	NA	NA	Yes	Yes				Yes	Yes	Yes	Yes	Yes
High-pitched Voice										Yes		
Expressive Language Delay	Yes	Yes	Yes	Yes	Yes	Yes	Yes	Yes	Yes	Yes	Yes	Yes
Mild to Moderate Intellectual Disability	Yes	Yes	Yes	Yes		Yes	Yes	Yes	Yes	Yes	Yes	Yes
Congenital Heart Defects	NA	NA			NA	Yes	Yes			NA	Yes	NA
Gastrointestinal Motility Issues	NA	NA		Yes				Yes	Yes		Yes	Yes
Behavioral Issues	Yes		Yes					Yes	Yes	Yes	Yes	

*Blank* feature absent, *NA* data not available

**Table 2:** Frequency of different clinical features in Chinese and Western FHS patients

Clinical Features	Frequency in present study (%)	Frequency reported (%)
Short Stature	12/12 (100.0)	59/70 (84.3)
Delayed Bone Age	10/10 (100.0)	38/68 (55.9)
Microcephaly	6/7 (85.7)	10/67 (14.9)
Short Neck	6/12 (50.0)	8/U
Triangular Face	8/12 (66.7)	61/64 (95.3)
Eyes		
• Deep-set Eyes	4/12 (33.3)	59/61 (96.7)
• Strabismus	4/12 (33.3)	8/46 (17.4)
• Long Eyelashes	9/12 (75.0)	58/59 (98.3)
Nose		
• Large Nares	9/12 (75.0)	2/U
• Low-hanging Columella	7/12 (58.3)	70/70 (100.0)
Mouth		
• Thin Upper Vermilion Border	8/12 (66.7)	67/67 (100.0)
• Wide Mouth	3/12 (25.0)	19/62 (30.6)
• Short Philtrum	7/12 (58.3)	65/69 (94.2)
Ears		
• Large Ears	8/12 (66.7)	3/U
• Low Set Ears	7/12 (58.3)	54/56 (96.4)
• Ear Deformity	7/9 (77.8)	NA
Dental Issues		
• Small Teeth/Widely Spaced Teeth	7/10 (70.0)	15/41 (36.6)
• Malocclusion/Underbite	3/12 (25.0)	4/43 (9.3)
• Cavities	3/8 (37.5)	6/40 (15.0)
Voice and Language		
• Hypernasality	3/12 (25.0)	9/U
• Expressive Language Delay	12/12 (100.0)	68/69 (98.6)
Feet and hands		
• Broad Thumbs	6/12 (50.0)	12/27 (44.4)
• Brachydactyly	8/12 (66.7)	14/U
• Broad Fingertips	4/12 (33.3)	4/U
• Clinodactyly V finger	4/12 (33.3)	13/U
Congenital Heart Defects	3/7 (42.9)	4/54 (7.4)
Genitourinary		
• Micropenis	2/6 (33.3)	NA
• Small Testis	1/6 (16.7)	NA
Gastrointestinal Motility Issues	5/10 (50.0)	14/54 (25.9)
Mild to Moderate Intellectual Disability	11/12 (91.7)	45/56 (80.4)
Behavioral Issues	6/12 (50.0)	27/60 (45.0)

*NA* data not available, *U* denominator unknown

### Facial gestalt

Our patients presented with similar facial features to Western FHS patients, including triangular face (8/12), long eyelashes (9/12), deep-set eyes (4/12), low set ears (7/12), low-hanging columella (7/12), short philtrum (7/12), thin upper vermilion border (8/12) and small and widely spaced teeth (7/10).

### Skeletal

Brachydactyly were noticed in eight of 12 individuals indicating it is a frequent feature in Chinese FHS patients. The frequency of broad thumbs in Chinese FHS patients (6/12) was similar to that of Western patients (12/27). Other skeletal features including clinodactyly V finger (4/12) and broad toes (4/12) were also found in our patients. In addition, some rare features were seen in our cohort including cubitus valgus (patient 3), middle phalange dysplasia (patient 6), pectus excavatum, genu varum and costal margin eversion (patient 8).

### Growth and bone age

The growth details for our cohort were summarized in Additional file 2: Table S1. All 12 patients in our cohort were described with short stature. Bone age was determined in ten patients and all patients presented with bone age delay.

### Endocrine findings

Ten patients (patient 2 and 7 excluded) underwent growth hormone stimulation tests by simultaneous administration of arginine (0.5 g/kg, intravenously) and clonidine (4 μg/kg, orally) (Additional file 2: Table S1). GH levels were measured at standard intervals during the tests (0, 30, 60, 90, 120 min). The GH peak levels of patient 3 and patient 5 were 8.65 ng/ml and 5.0 ng/ml respectively, suggesting partial growth hormone deficiency. Because the test for patient 5 was performed only by arginine along, her GH stimulation test result remained as undetermined. The other eight patients showed normal levels of growth hormone (Additional file 2: Table S1). In addition, among four individuals who underwent MRI scanning, two revealed a small but structurally normal pituitary gland (patient 1: 2.6 mm; patient 6: 2.5 mm) and patient 7 presented with a thin low part of the pituitary gland and no signal for the posterior part of the gland.

### Language, cognition and behavior issues

All 12 patients presented with severe expressive language delay. Their receptive language were much better than expressive language.

Eleven of 12 individuals showed intellectual disability, mostly of mild to moderate level. This is similar to those observed in Western patients (45/56). The Gesell developmental scale assessment for patient 1 revealed a adaptability development quotient (DQ) of 31, a gross motor DQ of 55, a fine motor DQ of 40, a language DQ of 14 and an individual communication DQ of 29. The score of Infant-Middle School Student Social Life Scale assessment was 6. The full scale IQ score using Chinese Wechsler intelligence scale for children (C-WISC) for patient 8 and patient 10 were 48 and 60, which indicated a moderate and mild intellectual disability respectively. Other nine patients were not performed with formal intellectual assessment.

Half of FHS patients (6/12) in this cohort exhibited behavior issues. Patient 1 attended regular school but did not interact with peers and may attack people if not satisfied. Patient 3 had learning difficulties. Inattention and skill regression were noticed in patient 8. Patient 9 presented with stereotype, attention deficit hyperactivity disorder, tantrums and impulsivity. Overdependence on mother and anxiety were also found in patient 10. Patient 11 also exhibited stereotypes and tantrums similar to patient 9.

### Genitourinary issues

We observed micropenis and/or small testis in 3 out of 6 male patients. Patient 1 (at 9 years and 2 months old) had small testes (1 ml of volume) and a thin penis (4 cm × 1.0 cm). Patient 2 (at 2 years and 2 months) and patient 7 (at one year) showed micropenis (2.5 cm × 0.8 cm) and (2.1 cm × 1.1 cm) respectively.

### Gastrointestinal issues

Five of ten patients who underwent gastrointestinal evaluation were found to have gastrointestinal issues. Patient 4 presented with symptoms of gastroesophageal reflex disease including hiccup and regurgitation after meals. The gastroesophageal reflux was also noticed in patient 8 and patient 11. Patient 11 also had constipation. Patient 9 and patient 12 showed gastrointestinal motility issues. Patient 12 was also found with Celiac disease.

### Cardiac malformation

Three of seven individuals who underwent cardiac evaluation showed congenital heart defects. Patient 6 had atrial septal defect, patent foramen ovale and persistent left superior vena cava. Patient 7 had mild aortic tricuspid valve insufficiency. In addition, patient 11 had a history of ventricular septal defect.

### Other rare features

Some rare findings were seen only once in our FHS patients, which may be incidental occurrence or likely novel phenotype for FHS. Patient 2 presented with neonatal pneumonia, agranulocytosis, hydrocele of tunica vaginalis and oblique inguinal hernia. Febrile convulsion occurred once at the age of three months in patient 3 and he also showed Cafe-au-Lait spots in the hypogastrium and lower limbs.

### Genetic analysis

We identified five different variants in 12 patients, two were previously reported (c.7303C > T/p.Arg2435Ter and c.7330C > T/p.Arg2444Ter) and the other three were novel (c.7189G > T/p.Glu2397Ter, c.7245_7246delAT/p.Ser2416ArgfsTer26 and c.7466C > G/p.Ser2489Ter) which were not described in common population databases (gnomAD, ExAC,1000 Genomes Project and Exome Variant Server) and germline variant databases (Human Gene Mutation Database, ClinVar and Leiden Open Variation Database). They are truncating(nonsense or frameshift)variants located in exon 34 resulting in loss of three C-terminal AT-hook motifs and all variants were proven to be de novo by parental Sanger sequencing (Table 1 and Additional file 1: Figure S1). According to the ACMG/AMP guidelines, these three novel variants were classified as likely pathogenic (PM1 + PM2 + PM6 + PP4)(PM:pathogenic moderate; PP: pathogenic supporting).

## Discussion

So far most FHS patients were reported in Western populations [4, 5, 15]. Few Chinese FHS patients had been reported [12]. This study represented the first cohort of Chinese FHS patients ascertained from multiple institutions across China.

This study discovered three novel likely pathogenic variants (c.7189G > T/p.Glu2397Ter, c.7245_7246delAT/p.Ser2416ArgfsTer26 and c.7466C > G/p.Ser2489Ter), which broadened *SRCAP* mutation spectrum. The c.7330C > T/p.Arg2444Ter mutation was the most common pathogenic variant so far detected among FHS patients from various ethnics groups [7]. The second most recurrently reported mutation in western population c.7303C > T/p.Arg2435Ter appeared to be more common in Chinese FHS patients [7]. All of the reported *SRCAP* mutations are nonsense or frameshift heterozygous variants located in a small region of exon 34, except for a stop mutation in exon 33 in two cases [5, 6]. They are predicted to cause a truncated SRCAP protein lacking the putative C-terminal AT-hook DNA binding motif [12], presumably escaping nonsense-mediated mRNA decay. The mechanism of disease has been postulated to be dominant-negative due to the non-random clustering of truncating mutations in the final exon that result in the loss of the major transactivation function of *SRCAP* located in a 655 residue C-terminal fragment [22]. The novel mutations discovered in Chinese patients are consistent with the disease mechanism in terms of mutation type and distribution.

Short stature is the most prominent feature of FHS. Growth hormone deficiency had been proposed as a possible cause of this phenotype, yet there was little data regarding GH levels in FHS patients. So far only two clinically diagnosed and none of molecularly confirmed FHS patients were reported to have GH deficiency [23, 24]. We identified the first molecularly confirmed FHS patient who showed partial growth hormone deficiency (patient 3) in China. A larger cohort study will be needed to determining the GH deficiency rate among FHS patients. In this study, eight patients underwent growth hormone treatment including patient 3 with partial hormone deficiency. At the last evaluation, the first-year delta height standard deviation score (SDS)in four patients(Patient 7–9 and Patient 11)was larger than 0.3, of those, three presented with annualized height SDS larger than 0.3 during the treatment, we defined these as of good response (Table 3). Although the first-year HV increase was larger than 3 cm/year in patient 8 (~ 3.6 cm/year), but his annualized height SDS was not as high (0.22 SD), we regarded him as of modest response to GH treatment. The first-year delta height SDS of patient 3 and patient 10 were less than 0.3, they were defined as of poor response. The remaining two patients just started GH treatment and the response is yet to be assessed. 14 molecularly confirmed FHS patients had been reported to undergo GH treatment previously [4, 5, 15, 16], but the effectiveness was rarely assessed or reported. It was reported that three patients exhibited good response [5] and one with poor response [16], the rest had insufficient information for assessing the response. Here we showed that about half of FHS patients in our cohort exhibited modest to good response to GH treatment. Our data demonstrated an general positive effect of GH treatment on height improvement for FHS patients. Yet the responsiveness was difficult to predict based on the GH levels or mutation types. Three patients (patient 3, patient 8 and patient 9) had the same mutation but their response were different. A limited mutation spectrum of FHS and a variable responsiveness [5, 23, 25–28] did not support a genotype-phenotype correlation in terms of GH treatment response for FHS patients. The longest duration of GH treatment in our cohort was 4 years and 3 months. A previously reported FHS patient underwent a 12-year GH treatment, his final height was 155 cm (− 2.83 SD) [28]. The long-term benefit is yet to be demonstrated for FHS patients using GH treatment.

**Table 3:** Summary of the Growth Hormone Treatment in 8 Chinese FHS patients

	Patient 3	Patient 6	Patient 7	Patient 8	Patient 9	Patient 10	Patient 11	Patient 12
Gender	Male	Female	Male	Female	Male	Male	Female	Female
*SRCAP* Mutation	c.7330C > T /p.Arg2444Ter	c.7330C > T /p.Arg2444Ter	c.7245_7246delAT/p.Ser2416ArgfsTer26	c.7330C > T /p.Arg2444Ter	c.7330C > T /p.Arg2444Ter	c.7303C > T /p.Arg2435Ter	c.7303C > T /p.Arg2435Ter	c.7466C > G /p.Ser2489Ter
GHD	Yes	No	ND	No	No	No	No	No
Initial age of treatment	5Y2M	2Y	4Y10M	5Y	6Y6M	5Y2M	1Y5M	2Y3 M
Height at the initiation of treatment (SDS)	92.5 cm (−4.52)	74.7 cm (−3.62)	92.3 cm (−4.12)	93.8 cm (−3.84)	95 cm (−5.30)	92.7 cm(−4.47)	68.5 cm (−4.17)	75.0 cm (− 4.17)
HV before treatment (cm/year)	4.5	NA	NA	~ 3.1	~ 3	4.5	NA	NA
Dosage & Usage	0.15 IU/kg.p.d (short-acting)	1.2 IU/kg.p.d (short-acting)	1.18 ~ 1.4 IU/kg.p.w (long-acting)	0.17 mg/kg.p.w (long-acting)	0.2 IU/kg.p.d (short-acting)	0.14~0.17 IU/kg.p.d (short-acting)	0.13~0.2 IU/kg.p.d (short-acting)	0.125 IU/kg.p.d (short-acting)
Duration of treatment	1Y	1 M	4Y + 2 M	1Y + 7 M	4Y + 3 M	4Y + 3 M	2Y + 2 M	3 M
Height at one year treatment(SDS)	98 cm (−4.40)	na	99.5 cm (−3.76)	100.5 cm (− 3.50)	101.9 cm (− 4.78)	98 cm (− 4.40)	87 cm (−2.77)^a^	na
Height at last examination(SDS)	98 cm (− 4.40)	na	120.5 cm (−2.56)	103.4 cm (− 3.49)	119 cm (− 3.86)	118 cm (− 3.31)	88.6 cm (− 2.96)	77.9 cm (− 3.81)
First-year delta height SDS	0.12	na	0.36	0.34	0.52	0.07	> 0.7	na
Total height SDS	0.12	na	1.56	0.35	1.44	1.16	1.21	na
Annualized height SDS	0.12	na	0.37	0.22	0.34	0.27	0.56	na
HV during the first year of treatment (cm/year)	5.5	na	6.64	6.7	6.9	5.3	10.1	na
HV increase during the first-year (cm/year)	1	na	na	~ 3.6	~ 3.9	0.8	na	na
GH Treatment response	Poor	ND	Good	Modest	Good	Poor	Good	ND

*ND* undetermined, *na* not applicable, *NA* data not available, a: data collected at one year and ten months after GH treatment

We compared the clinical presentations of Chinese FHS patients with common features of this condition based on patients of other ethnic backgrounds (Table 2). The shared features include short stature, delayed bone age, broad thumbs, language deficits, mild to moderate intellectual disability, gastrointestinal motility issues, behavioral issues and facial dysmorphic features. The main common facial features are triangular face, long eyelashes, deep-set eyes, low set ears, low-hanging columella, short philtrum, thin upper vermilion border, small and widely spaced teeth. Interestingly, we noticed that at least half (> 6/12) of our patients had microcephaly (one patient had a normal OFC, we did not have data for the other five patients). The frequency of microcephaly in this Chinese cohort is much higher than that of Western patients (10/67). In addition, we reported minor ear anormalies in 7/9 patients (Additional file 2: Table S1). Those features were also frequently observed in previously published patients but it was not specifically mentioned. We think although the deformities were not major and variable between individuals, the ear phenotypes of FHS patients are worth paying attention to. Furthermore, we described micropenis and/or small testis in 3 out of 6 male patients. Cryptorchidism and hypospadia had been reported as recurrent features of FHS patient [7]. Micropenis and small testis may represent novel features for a subset of FHS patients. A large cohort will be needed to determining if features of hypogonadism is a recurrent phenotype associated with FHS patients and how they may affect their reproductive potential.

Although not a very large cohort, the comprehensive evaluation of dysmorphological features of our patients offered the first opportunity to compare the differences between Chinese and Western FHS patients. As showing in Table 2, the following facial features exhibited significant differences in Western vs. Chinese patients: triangular face (95.3% vs. 66.7%), short philtrum (94.2% vs 58.3%), deep-set eyes (96.7% vs. 33.3%), long eyelashes (98.3% vs. 75%), low-hanging columella (100% vs. 58.3%) and thin upper vermilion border (100% vs. 66.7%). These differences may be attributable to intrinsic ethnic difference, it may also due to the fact that most of our patients were diagnosed by whole exome sequencing whereas most patients in Western countries were ascertained initially based on typical presentations of clinical features. Genotype-first diagnosis is revealing more atypical cases of classic syndromes [29].

In addition, some previously reported features were not observed in our patients. These features include cleft lip/pseudocleft lip, high-arched palate, velopharyngeal insufficiency, hyperopia, recurrent otitis media, astigmatism, conductive hearing loss, clavicular pseudarthrosis, clavicular hypoplasia, hip dysplasia, scoliosis/kyphosis, Renal/collecting system anomalies, hydronephrosis, polycystic kidneys, seizures, Legg-Calvé-Perthes disease, syndactyly, hirsutism, hypertension, oligodontia and supernumerary maxillary teeth [6–8, 15, 16, 23, 30]. These features are not common and they may not be observed in a small patient cohort.

## Conclusions

In this study, we delineated the genotypes and phenotypes of 12 Chinese FHS patients, which broaden the *SRCAP* mutation spectrum and clinical phenotype spectrum of FHS. The modest to good responses of our patients who underwent GH treatment help to demonstrate the overall benefit of this treatment. Longer term follow up and more patient evaluation will be needed to better understand this condition and the prognosis.

## Materials and methods

### Subjects

All individuals were ascertained by molecular diagnostic test due to short stature of uncertain reason. Short stature is defined as a height which is 2 standard deviations below the mean height of the Chinese population with the same sex and age. Twelve individuals were from The Maternal and Child Health Hospital of GuangXi Zhuang Autonomous Region, Children’s Hospital of Chongqing Medical University, Jiangxi Provincial Children’s Hospital, Shandong Provincial Hospital, KingMed Diagnostics, The First Affiliated Hospital of Sun Yat-sen University, XI’AN Children’s Hospital, GUIZHOU Provincial People’s Hospital and The Second Hospital of Hebei Medical University. Approval of the study design was in compliance with the Helsinki Declaration and was obtained from each of the participating institutions’ review boards. Informed consent was obtained from each study subject (or their guardian) prior to enrollment. In most cases, clinical photographs were available.

### Molecular analysis

Peripheral venous blood samples with EDTA anti-coagulant were collected from the patients and their parents. Genomic DNA was extracted from peripheral blood leukocytes using QIAamp DNA Blood Mini Kit (Qiagen, Germany) according to the manufacturer’s instructions. The major steps of whole exome sequencing were as described below: Library preparation, cluster generation and sequencing were performed according to the manufacturer’s protocols. Library preparation for WES was performed using the Agilent SureSelect Human All Exon kit V5 (Agilent, Santa Clara, CA). Bcl2fastq tool (v2.15.0.4) was used for extracting Fastq files from Illumina bcl sequencing file. BWA (0.7.10-r789), Picard (v1.128) and Genome Analysis Toolkit (GATK v3.5) were employed for genome alignments and variant detection. The Annovar tool was applied for variant annotation. Common variants were filtered based on their frequencies in the databases of the Exome Aggregation Consortium (ExAC) (http://exac.broadinstitute.org), the Exome Sequencing Project (https://esp.gs.washington.edu), or 1000G (http://www.1000genomes.org), and our internal database. The pathogenicity of the sequence variants was interpreted according to the American College of Medical Genetics and Genomics/Association for Molecular Pathology (ACMG/AMP) guidelines [31]. All putative pathogenic variants detected in the patients by WES were confirmed by Sanger sequencing (Additional file 1: Figure S1). Parents of probands were performed Sanger sequencing of the detected mutation, which was used to identify the origins of the variants.

### Growth hormone stimulation tests

Growth hormone stimulation tests was performed by two kinds of drugs (clonidine 4 μg/kg, orally, and arginine 0.5 g/kg, intravenously). Blood samples were collected to determine baseline levels of insulin-like growth factor 1 (IGF-1). GH levels were measured at standard intervals during the tests (0, 30, 60, 90, 120 min). According to current guidelines [32], the peak growth hormone levels between 5 and 10 ng/ml on stimulation testing are defined as partial growth hormone deficiency, the peak growth hormone levels less than 5 ng/ml are defined as complete growth hormone deficiency and peak growth hormone levels more than 10 ng/ml are defined as normal.

### Defining growth response of GH treatment

In this study, we calculated height SDS based on Chinese growth curves [33]. To evaluate the growth response of GH treatment, we mainly used height SDS (including the first-year and annualized height SDS) and referred to height velocity. We regarded patients with a first-year delta height SDS larger than 0.3–0.5 or a first-year height velocity increase larger than 3 cm/year as of good response [34]. If the annualized height SDS of these patient was smaller than 0.3, it was defined as of modest response. If the response was worse than above measurements, it was defined as of poor response. Patients treated for less than one year were not evaluated.

## Additional files

Additional file 1: **Figure S1.** The Sanger sequencing results of 12 FHS patients. (TIF 1342 kb)

Additional file 2: **Table S1.** Clinical details of 12 FHS patients in our cohort. (DOCX 19 kb)

## Data Availability

The datasets used and analysed during the current study are available from the corresponding author on reasonable request.

## Abbreviations

ACMG/AMP: American College of Medical Genetics and Genomics/Association for Molecular Pathology
BA: Bone Age
CA: Chronological Age
CREBBP: CREB-binding protein
C-WISC: Chinese Wechsler intelligence scale for children
DQ: Development quotient
ExAC: Exome Aggregation Consortium
FHS: Floating-Harbor syndrome
GATK: Genome Analysis Toolkit
GH: Growth hormone
GHD: Growth Hormone Deficiency
HGMD: Human Gene Mutation Database
HV: Height Velocity
IGF-1: Insulin-like growth factor 1
RTS: Rubinstein-Taybi syndrome
SDS: Standard Deviation Score
WES: Whole exome sequencing
