# Supplementary figures and images for: Novel genotypes and phenotypes among Chinese patients with Floating-Harbor syndrome
Source: Orphanet J Rare Dis. 2019 Jun 14;14:144. doi: 10.1186/s13023-019-1111-8 (PMC6570847; doi:10.1186/s13023-019-1111-8)

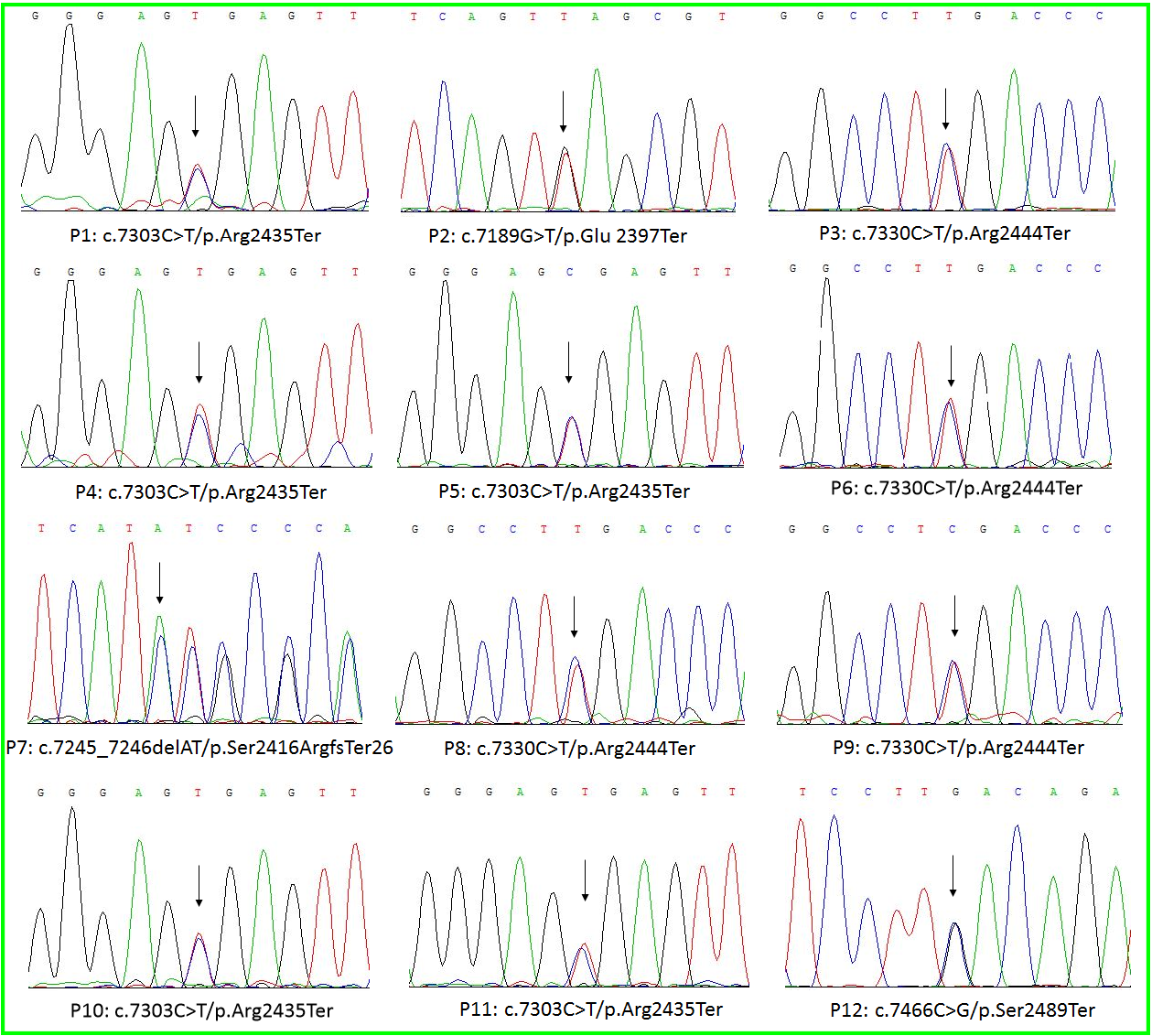

Supplement: Supplementary file 1 — Figure S1. The Sanger sequencing results of 12 FHS patients. (TIF 1342 kb) [file 13023_2019_1111_MOESM1_ESM.tif]
