# Supplementary material for: Novel genotypes and phenotypes among Chinese patients with Floating-Harbor syndrome
Source: Orphanet J Rare Dis. 2019 Jun 14;14:144. doi: 10.1186/s13023-019-1111-8 (PMC6570847; doi:10.1186/s13023-019-1111-8)
Supplement: Supplementary file 2 — Table S1. Clinical details of 12 FHS patients in our cohort. (DOCX 19 kb) [file 13023_2019_1111_MOESM2_ESM.docx]

Table S1. Clinical details of 12 FHS patients in our cohort.

|  | **Patient 1** | **Patient 2** | **Patient 3** | **Patient 4** | **Patient 5** | **Patient 6** | **Patient 7** | **Patient 8** | **Patient 9** | **Patient 10** | **Patient 11** | **Patient 12** |
| --- | --- | --- | --- | --- | --- | --- | --- | --- | --- | --- | --- | --- |
| **Gender** | Male | Male | Male | Female | Female | Female | Male | Female | Male | Male | Female | Female |
| **Birth Date** | 2005.4.30 | ~2015.4 | 2008.9 | 2011.12.4 | ~2015.4 | 2016.11.13 | 2009.10.31 | 2012.6.9 | 2005.10.7 | 2009.8.13 | 2015.5.7 | 2016.5.30 |
| **Gestation** | 36w | NA | 32w | Full Term | 38w | 38w | 42w | 38w | Full Term | NA | 40w+3d | 37w+4d |
| **History of Gestation** | G1P1 | G2P2 | G3P3 | G1P1 | NA | G4P2 | G2P2 | G1P1 | NA | adopted child | G2P1 | G2P2 |
| **Birth Length** | NA | NA | NA | NA | 50.6cm | 40cm | 46cm | NA | 49cm | NA | NA | 49cm |
| **Birth Weight** | 2.3kg | 2.6kg | 2.5kg | 2.6kg | NA | NA | 2.4kg | 2.75kg | 2.8kg | NA | NA | 2.6kg |
| **Low Birth Weight** | Yes |  |  |  | NA | NA | Yes |  |  | NA | NA |  |
| **Age at first**  **assessment** | 9Y2M | 2Y2M | 5Y2M | 5Y6M | 1Y6M | 2Y | 1Y | 5Y | 6Y6M | 5Y2M | 1Y5M | 2Y2M |
| **Height** | 95.8cm  /-6.86SD | 78cm  /-3.44SD | 92.5cm  /-4.52SD | 99.1cm  /-3.26SD | 74.5cm  /-2.31SD | 74.7cm  /-3.62SD | 67cm  /-3.48SD | 93.8cm  /-3.84SD | 95cm  /-5.30SD | 92.7cm  /-4.47SD | 68.5cm  /-4.17SD | 74.3cm  /-4.20SD |
| **Weight** | 10kg  /-4.59SD | 8.2kg  /-3.84SD | NA | NA | NA | NA | 8.5kg  /-1.50SD | 11kg  /-4.0SD | NA | NA | NA | 8kg  /-3.74SD |
| **OFC** | 46cm  /<-4.38SD | 46cm  /-2.08SD | NA | NA | NA | 42.5cm  /-3.84SD | 43cm  /-2.62SD | 46.5cm  /-2.78SD | 52cm  /-0.23SD | NA | NA | 44cm  /-2.80SD |
| **Parents Information** | F:165cm(36)  M:160cm(30) | F:173cm  M:157cm | F:167cm(38)  M:165cm(38) | F:173cm  M:158cm | F:(31)  M:(28) | F:172cm(28)  M:168cm(27) | F:171cm(30)  M:156cm(24) | F:178cm(34)  M:167cm(32) | F:163cm(33)M:152cm(36) | NA | F:170cm(32)  M:162cm(24) | F:168cm(30)  M:157cm(30) |
| **Significant Delayed Bone Age** | BA=2Y8M  /CA=9Y2M | BA=3M  /CA=2Y2M | BA=3Y5M  /CA=5Y2M | BA=2Y  /CA=5Y6M | NA | BA=6~9M  /CA=2Y | BA=2Y9M  /CA=5Y10M | BA=2Y6M  /CA=5Y9M | BA=2Y6M  /CA=8Y8M | BA=5Y  /CA=8Y5M | NA | BA=1Y1M  /CA=2Y6M |
| **Short Neck** |  | Yes |  | Yes | Yes |  |  | Yes |  | Yes |  | Yes |
| **Broad Chest** |  |  |  | Yes |  |  |  |  | Yes |  |  |  |
| **GH Peak (ng/ml)** | 10.47 | NA | 8.65 | 22.9 | 5.0 | 10.49 | NA | 12.2 | 10.56 | 14.6 | 11.5 | 13.2 |
| **IGF-1(ng/ml)** | 97.6 | NA | NA | 69.76 | 57.8 | 49.2 | NA | 163 | 343 | 141 | 56.5 | 86.5 |
| **GHD** |  | ND | Yes |  | ND |  | ND |  |  |  |  |  |
| **Hypotonia** |  |  |  |  |  | Yes |  | Yes |  |  |  |  |
| **Downslanting Palpebral Fissures** |  |  |  |  |  |  |  |  |  |  | Yes |  |
| **Strabismus** | Yes |  | Yes |  |  | Yes |  |  | Yes |  |  |  |
| **Visual Impairment** |  |  |  |  |  |  |  |  | Yes |  |  |  |
| **Photophobia** |  |  |  |  |  |  |  |  | Yes |  |  |  |
| **Large Nares** | Yes |  | Yes |  |  | Yes | Yes | Yes | Yes | Yes | Yes | Yes |
| **Large Ears** | Yes |  |  |  | Yes |  | Yes | Yes | Yes | Yes | Yes | Yes |
| **Posteriorly Rotated Ears** |  | Yes |  |  |  |  |  |  |  |  |  |  |
| **Protruding Ears** |  |  |  |  |  |  |  |  |  |  | Yes | Yes |
| **Ear Deformity** |  | NA | Yes^a^ | Yes^b^ | NA | NA |  | Yes^c^ | Yes^d^ | Yes^e^ | Yes^f^ | Yes^g^ |
| **Low Anterior Hairline** |  |  |  |  |  |  |  |  |  |  | Yes |  |
| **High Frontal Hairline** |  |  |  |  |  |  |  |  |  | Yes |  |  |
| **Low Posterior Hairline** |  | Yes |  | Yes |  |  |  |  |  |  |  |  |
| **Malocclusion**  **/Underbite** |  |  |  |  |  |  |  | Yes | Yes | Yes |  |  |
| **Cavities** | NA | NA |  | NA |  | NA |  | Yes | Yes | Yes |  |  |
| **Micrognathia** |  |  |  |  | Yes |  |  |  |  |  |  |  |
| **Prominent Forehead** |  | Yes |  |  |  |  |  |  |  |  |  |  |
| **Hypernasality** |  |  |  |  |  |  | Yes |  |  | Yes | Yes |  |
| **Broad Toes** |  |  | Yes |  |  |  |  |  | Yes |  | Yes | Yes |
| **Brachydactyly** |  |  | Yes | Yes |  | Yes |  | Yes | Yes | Yes | Yes | Yes |
| **Broad Fingertips** |  |  |  | Yes |  |  |  | Yes | Yes |  |  |  |
| **Finger Clubbing** |  |  | Yes |  |  |  |  | Yes | Yes |  |  |  |
| **Hypoplastic or Absent Nails** |  |  |  |  |  | Yes |  |  | Yes |  |  |  |
| **Joint Hyperlaxity** |  |  |  |  |  |  |  | Yes |  |  |  |  |
| **Micropenis** |  | Yes |  |  |  |  | Yes |  |  |  |  |  |
| **Small Testis** | Yes |  |  |  |  |  |  |  |  |  |  |  |
| **Celiac Disease** | NA | NA |  |  |  |  |  |  | NA | NA | NA | Yes |
| **Other features** | poor appetite | motor delay |  |  |  | motor delay；  single palmar crease； | SGA；reduced fetal movement | psychomotor delay;  Babinski sign(+);  Knee-jerk Reflex(-);  high ACTH;  highcortisol; |  |  |  |  |

Blank: feature absent; ND: undetermined; NA: data not available; a:asymmetrical ears; deformed auride on left ear; angulated antihelix; bilateral ear lobe creases; b:underdeveloped antihelix c:angulated antihelix on right ear d:prominent antihelix; e:bilateral prominent antihelix; f:bilateral prominent antihelix; g:ear lobe crease on left ear; SGA: small for gestation; ACTH: adrenocorticotrophic hormone.
